# Novel Conductive AgNP-Based Adhesive Based on Novel Poly (Ionic Liquid)-Based Waterborne Polyurethane Chloride Salts for E-Textiles

**DOI:** 10.3390/polym16040540

**Published:** 2024-02-17

**Authors:** Haiyang Liao, Yeqi Xiao, Tiemin Xiao, Hongjin Kuang, Xiaolong Feng, Xiao Sun, Guixin Cui, Xiaofei Duan, Pu Shi

**Affiliations:** 1 School of Mechanical Engineering, Hunan University of Technology, Zhuzhou 412007, China; haiyangliao1990@163.com (H.L.);; 2 China Textile Academy (Zhejiang) Technology Research Institute Co., Ltd., Shaoxing 312071, China

**Keywords:** ionic waterborne polyurethane, AgNP-based conductive adhesive, low-temperature sintering, e-textile

## Abstract

The emergence of novel e-textile materials that combine the inherent qualities of the textile substrate (lightweight, soft, breathable, durable, etc.) with the functionality of micro/nano-electronic materials (conductive, dielectric, sensing, etc.) has resulted in a trend toward miniaturization, integration, and intelligence in new electronic devices. However, the formation of a conductive network by micro/nano-conductive materials on textiles necessitates high-temperature sintering, which inevitably causes substrate aging and component damage. Herein, a bis-hydroxy-imidazolium chloride salt as a hard segment to synthesize a waterborne polyurethane (WPU) adhesive is designed and prepared. When used in nano-silver-based printing coatings, it offers strong adherence for coatings, reaching 16 N cm^−1^; on the other hand, the introduction of chloride ions enables low-temperature (60 °C) chemical sintering to address the challenge of secondary treatment and high-temperature sintering (>150 °C). Printed into flexible circuits, the resistivity can be controlled by the content of imidazolium salts anchored in the molecular chain of the WPU from a maximum resistivity of 3.1 × 10^7^ down to 5.8 × 10^−5^ Ω m, and it can conduct a Bluetooth-type finger pulse detector with such low resistivity. As a flexible circuit, it also offers high stability against washing and adhesion, which the resistivity only reduces less than 20% after washing 10 times and adhesion. Owing to the adjustability of the resistivity, we fabricated an all-textile flexible pressure sensor that accurately differentiates different external pressures (min. 10 g, ~29 Pa), recognizes forms, and detects joint motions (finger bending and wrist flexion).

## 1. Introduction

Smart textiles are an appealing field to investigate because of the rapid growth of printed electronics and their application in hot areas such as wearable electronics, robotic systems, and motion monitoring [1,2,3,4,5,6,7]. Textile-based wearable electronics are a key part of smart textiles [8]. The softness and skin-friendly properties of textiles allow wearable electronics to monitor movement more accurately, making smart textiles a great candidate for application as flexible strain sensors and conductive textiles [9,10,11,12]. Current approaches to rendering textiles conductive, such as implanting metal wires and co-woven carbon fibers, are incompatible with the substrate, and there is even a risk of puncturing the fabric after a prolonged period [13,14,15]. Thus, understanding how to make the textiles perform as ongoing and stable conductive materials has become a challenge.

Metallic nanoparticles with high electrical conductivity and high stability, such as silver nanoparticles (AgNPs), have attracted great interest in flexible electronics [16,17,18,19]. The stable dispersion of AgNPs in various solvents makes it very easy to generate a stable conductive ink, and the AgNPs have high compatibility with polymer-based adhesives (epoxy-based, acrylic-based, polyurethane-based, etc.) [20,21,22]. Such adhesives, especially waterborne polyurethanes (WPUs), offer high flexibility and stickiness, which considerably aids in the attachment and durability of nanoparticles to the substrate [23,24]. The simplest method to fabricate flexible e-textiles is to print AgNP-based conductive coatings with WPU adhesive directly on the fabric surface. However, the printed AgNP coatings do not actually conduct electricity because the AgNP surface is absorbed by a layer of non-conductive stabilizer (sodium citrate, polyvinylpyrrolidone, cetylammonium bromide, etc.) [25]. Generally speaking, it is necessary to use the second high-temperature sintering (more than 150 °C) step to remove the surface stabilizers and sinter the material into a dense block to form a conductive network; the use of high temperatures that are not conducive to the stability of the fabric substrate will inevitably lead to shrinkage or mechanical problems in the attenuation [26,27,28]. To maintain the benefits of textile substrates, it is critical to develop a low-temperature/room temperature sintering technique for activating the conductive network of AgNPs.

Research has reported that the addition of Cl^−^ to AgNP coatings induces the surface stabilizers to dissociate, allowing for low-temperature sintering [29,30]. Unfortunately, a strong Lewis acid of Cl^−^ is detrimental to the stabilization of WPUs and may cause them to flocculate; on the other hand, they are unable to penetrate into the molecular chain of WPUs [31,32]. Ionic liquids (ILs) are organic salts composed entirely of cations and anions with a range of unique properties such as low vapor pressure, non-flammability, non-volatility, and excellent chemical stability. In addition, they have highly functional design capabilities such as hydroxylation, amination, carboxylation, anion tunability, etc. It is believed that designed IL molecules can be anchored in the WPU chain to overcome ion permeation and instability challenges. In this paper, a bis-hydroxyimidazole chloride salt is synthesized by a quaternization reaction. The molecular structure can react with isocyanate groups (-NCO) to introduce the imidazolium chloride salt into the molecular structure of the WPU, allowing for low-temperature sintering of conductive coatings for AgNPs without the addition of chloride salt. The resistivity of AgNP conductive coatings can be controlled by varying the content of imidazolium chloride salt (from 10^6^–10^−5^ Ω m). Depending on the resistivity change, it is possible to employ AgNP coatings as flexible printed circuits to link electronic components as wires, or as dielectric materials, to realize sensing capability for pressure measurement and motion capture. This ionic WPU_[Cl−]_ adhesive has the potential to be utilized in the production of e-textiles, as well as a binder in energy materials.

## 2. Experimental Section

### 2.1. Materials

Silver nitrate (AgNO_3_, 99.8%), ferrous sulfate heptahydrate (FeSO_4_ 7H_2_O, 99.8%), sodium citrate (CA-Na, Na_3_C_6_H_5_O_7_ 2H_2_O, 98%), ethanol (C_2_H_5_OH, 99.9%), glycerol (C_3_H_8_O_3_, 99.9%), N,N-Dimethylformamide (DMF, AR, 99%), and ethylene glycol (C_2_H_6_O_2_, 99.9%) were purchased from Beijing InnoChem Science & Technology CO. Ltd., Beijing, China. 1-(2-Hydroxyethyl) imidazole (AR) and 1,2-Dichloroethane (ACS, 99.0%) were purchased from Shanghai Aladdin Biochemical Technology Co., Ltd., Shanghai, China. 2,2-Dihydroxymethylpropionic acid (DMPA, 99.0%), isophorone diisocyanate (IPDI, 99.0%), dibutyltin dilaurate (DBTDL, 99%), and polytetrahydrofuran (PTMEG, Mn = 2000) were provided by Shanghai Macklin Biochemical Technology Co., Ltd., Shanghai, China. PET woven fabric (100 g m^−2^) was obtained from Dongguan Zhicheng Fiber Products Co., Dongguan, China. All chemical agents and materials were used as received without any other purification.

### 2.2. Preparation of AgNPs

The AgNPs were prepared by a liquid phase reduction method. Typically, 2.5 g of AgNO_3_ was dissolved in 22.5 mL of pure water, and recorded as solution A. An amount of 14 g of CA-Na was dissolved in 21 mL of pure water, and another 7.5 g of FeSO_4_ 7H_2_O was dissolved in 17.5 mL of pure water; subsequently, the two solutions were mixed and recorded as solution B. Solution B was slowly dropped into solution A for a sustained reaction of 1 h and then centrifuged in NaNO_3_ solution. The black precipitate was collected and dried in a vacuum for 12 h at 30 °C.

### 2.3. Preparation of Bis(Hydroxy)-Capped Imidazole Chloride Salt (OH-IL_[Cl-]_-OH)

Hydroxy-capped imidazole chloride salt was synthesized by quaternary nitridation reaction [33,34], as shown in Figure S1a. Typically, 1-(2-Hydroxyethyl) imidazole and 1,2-Dichloroethane were charged into a flask containing DMF, at a molar ratio of 1:1.15. The flask was kept under N_2_ at 80 °C for 72 h. Next, the reactants were precipitated with a large amount of ethyl acetate (more than three times the volume of the reaction solution), washed with anhydrous acetonitrile, and finally dried under vacuum at 50 °C for 12 h (1H-NMR, 500 MHz, DMSO, ppm, see Figure S1b: 9.5 (1H, s, CH), 8.2 (1H, s, CH), 7.7 (1H, s, CH), 5.5 (1H, s, OH), 4.6 (2H, s, CH_2_), 4.2 (2H, t, J = 7.8 Hz CH_2_), and 3.3 (2H, t, J = 7.5 Hz CH_2_)).

### 2.4. Preparation of Waterborne Polyurethane Adhesives Containing Chloride Ions (WPU_[Cl−]_)

The reaction flask was filled with PTMEG (0.02 mol) and DMPA (0.01 mol) and agitated at 120 °C for 2 h to remove moisture absorbed by the monomers. Following that, IPDI (keeping the molar ratio of -NCO to -OH functional group of 1.2:1) and DBTDL as the catalyst (80 mg) were added to the flask along with 10 mL of acetone; the reaction was carried out at 80 °C for 4 h. After the temperature fell to 60 °C, OH-IL_[CL-]_-OH (0, 0.002, 0.005, 0.008, and 0.01 mol) was injected into the reactor and the reaction was maintained for another 4 h. After cooling the reactor down to room temperature, 160 mL of water was added dropwise to the system and vigorously agitated until emulsification was obtained. To produce a salt-form reaction with the carboxylic group of DMPA, the emulsified polyurethane was neutralized with a NaOH solution (0.01 mol). Finally, a WPU_[Cl−]_ solution was prepared by rotary evaporation to remove the acetone.

### 2.5. Preparation of Printable AgNP-Based Flexible Circuits

**Figure 1:**
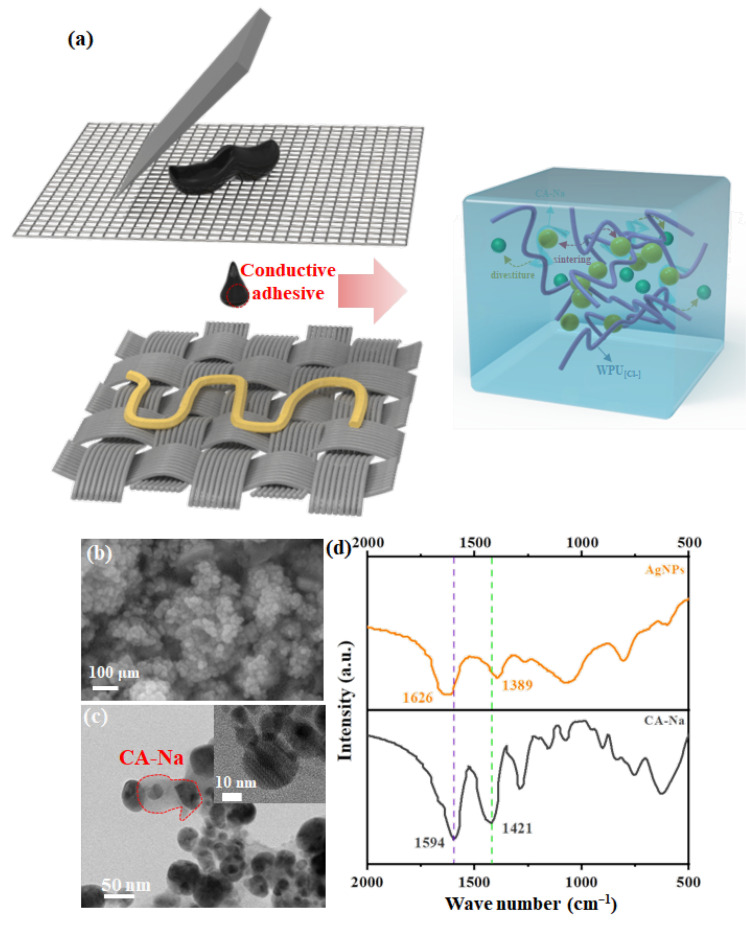
(**a**) Schematic illustration for fabrication of AgNP-based printed conductive pattern; (**b**) SEM image of the as-prepared AgNPs; (**c**) TEM image of as-prepared AgNPs and insert image shows the HR-TEM image; and (**d**) FTIR spectrum of CA-Na and AgNPs.

AgNPs were ultrasonically dispersed in a co-alcoholic solvent (pure water: ethanol: glycerol: ethylene glycol = 24:8:30:38, *v*/*v*) configured to form a 0.5 g mL^−1^ dispersion, which was subsequently mixed with an equal mass of WPU_[Cl−]_ to form an electrically conductive solution with a solid content of 60%. Woven polyester (PET, 2.3 cm × 3.6 cm × 100 μm, 150 g m^−2^) was first washed with ethanol and pure water and used as a substrate for screen printing. A 250-mesh nylon sieve was used for screening, and the conductive solution was uniformly applied to the mesh plate, ensuring that the angle between the squeegee and the mesh plate was 45° for printing. Finally, after 60 °C heat curing for 3 h, the printed flexible circuit pattern was obtained (Figure 1a).

### 2.6. Assembly of Flexible Pressure-Sensing Polyester Fabric (PET-FPS)

To assemble PET-FPS, two pieces of PET (2.3 cm × 2.3 cm × 125 μm) printed with a rectangular conductive pattern are stacked and organized together in a face-to-face manner, and then two pieces of aluminum foil paper are fixed with conductive silver paste at the end of the two pieces of conductive PET fabric, leaving a 1.5 cm pressure-sensing area as a lead, which can then be assembled into a simple resistive flexible pressure sensor.

### 2.7. Characterization

The Fourier-transform infrared spectroscopy (FTIR) method, employing a Nicolet 6700 spectrometer from Thermo Fisher Scientific, Co., Waltham, MA, USA, and the nuclear magnetic resonance spectroscopy (NMR) method, utilizing a Bruker AV 400 spectrometer from Bruker Co., Bremen, Germany, were utilized to investigate the chemical structure of both the monomers and the resultant hydrogels. The crystal structure of the AgNPs was recorded by X-ray diffraction (XRD, DMAX-Ultima IV, Rigaku Co., Tokyo, Japan). The morphology of the samples was observed by scanning electron microscopy (SEM; SN-3400, Hitachi Ltd., Tokyo, Japan) and a transmission electron microscope (TEM, JEM-2010F, JEOL Ltd., Tokyo, Japan). Nanoparticle size and zeta potential analyzers (DLS, ZS90, Malvern Panalytical Ltd., Malvern, UK) were employed to test the particle size of AgNPs and WPU_[Cl−]_ emulsions at a concentration of 2 wt.% dispersion. The tensile strength of the WPU_[Cl−]_ was determined using a mechanical tester (Zwick-Roell Z005, Ulm, Germany), with a strain rate of 100 mm min^−1^ and an initial gauge length of 40 mm. Thermogravimetric analysis (TGA; Q50, TA. Instruments Ltd., New Castle, DE, USA) was used to test information on thermal weight loss. The chemical component of the sample was analyzed by X-ray photoelectron spectroscopy (XPS; Thermo ESCALAB 250XI) with Al Kα as the X-ray source. The resistance signals were recorded by an LCR meter (E4980AL-102, Keysight Technologies Ltd., Santa Rosa, CA, USA) and monitored by a customized Keysight PathWave. The Δ*R/R_0_* of the deformed PET-FPS was calculated as Equation (1) [35,36]:

(1) $$∆R/R_{0}=\frac{\left|R-R_{0}\right|}{R_{0}}$$

where *R* is the real-time resistance and *R_0_* is the initial resistance.

## 3. Results and Discussion

### 3.1. Characterization of Raw Materials for AgNP-Based Conductive Adhesive

Sodium citrate as a salt stabilizer ionizes and binds to Ag^+^ in solution, and as Ag^+^ is reduced to atomic clusters by FeSO_4_ and grows into AgNPs, the negatively charged citrate is also adsorbed on the surface of the AgNPs, thus increasing its surface potential energy and preventing agglomeration between nanoparticles. In addition, in the TG test of AgNPs, it can be seen that there is a significant mass drop in AgNPs at 230 °C, after which the mass stays steady up to 800 °C without any significant drop (Figure S2). Calculation of the mass difference between them shows that the mass of the stabilizer adhering to the surface of AgNPs is ~1.2%. Confirming this view, the SEM image (Figure 1b) shows that the well-bounded AgNPs are uniformly dispersed. Further, as far as the particle size can be seen from the TEM image (Figure 1c), the AgNP morphology presents round particles with a size of only ca. 30 nm; moreover, the lattice stripes inside the particles are clearly visible and their spacing corresponds to the 111 crystallographic planes on the XRD (Figure S3), in addition to which there is also a layer of film-like material encapsulated on the surface of the particles. Further, by comparing the FTIR of CA-Na and AgNPs (Figure 1d), it can be seen that the symmetric and asymmetric vibrational absorption peaks of carboxylate in the pristine sodium citrate are located at 1594 cm^−1^ and 1421 cm^−1^, respectively, and the difference between the two is 173. Whereas the symmetric and asymmetric vibrational absorption peaks of carboxylate are found to be blue-shifted to 1626 cm^−1^ and 1389 cm^−1^ in the AgNPs and the difference increases to 237, indicating that the carboxylic acid in sodium citrate adsorbs on the surface of the AgNPs [37,38]. In terms of the WPU_[Cl−]_ adhesive (Figure S4), it is made of PETMG, DMPA, OH-IL_[Cl-]_-OH, and IPDI coupled with the reaction of -NCO with -OH. In this way, only the imidazole ring, ether bond, and ester bond become the characteristic peaks. The C=O characteristic band at 1726 cm^−1^ was observed, and the stretching vibrations are attributed to the C-N and C=N double bonds on the imidazole ring at 1661 and 1391 cm^−1^. C-O-C stretching vibrations at 1121 cm^−1^ were also observed, which suggests that all of these characteristic monomers on the WPU_[Cl−]_ adhesive were successfully coupled with the reaction of -NCO with -OH [39].

### 3.2. Stability and Print Quality of AgNP-Based Conductive Adhesive

**Figure 2:**
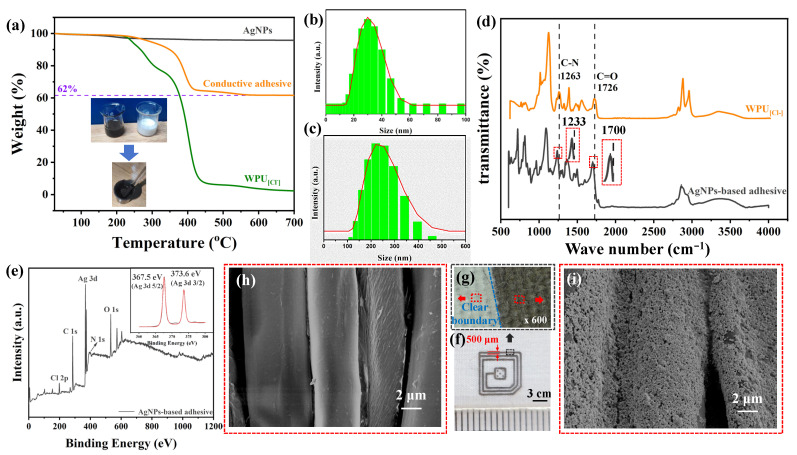
(**a**) TG curves of AgNPs and conductive adhesive and WPU_[Cl−]_; (**b**) particle size distribution of AgNPs; (**c**) particle size distribution of WPU_[Cl−]_; (**d**) FTIR spectra of conductive adhesive and WPU_[Cl−]_; (**e**) XPS spectra of AgNP-based conductive adhesive with 2 mmol of WPU_[Cl−]_; (**f**) photo of printed pattern on textile; (**g**) microscopic photograph of the demarcation line of a printed pattern; differences in the morphology of patterns printed on textile; (**h**) SEM image of unprinted site; and (**i**) SEM image of printed site.

Conductive AgNP-based adhesives with different contents of AgNPs can be obtained by homogeneously mixing AgNP co-ethanol solution with WPU_[Cl−]_. In order to accurately test the content of AgNPs in the conductive adhesive, the TG measurement was used to estimate the content of AgNPs based on the different thermal stability values of each component in the adhesive. The TG curves of both the conductive adhesive and WPU_[Cl−]_ exhibit sustained weight loss in the range of 100–400 °C, which is mainly due to the thermal decomposition of WPU_[Cl−]_. The content of the conductive adhesive can be estimated as 62 wt.% based on the difference in their weight loss (Figure 2a). On the other hand, the stability of the conductive adhesive is a guarantee of high-quality printing. Whether AgNPs can be uniformly dispersed in solution with WPU_[Cl−]_ is a prerequisite to guarantee the stability of the conductive adhesive. In order to investigate the dispersion of the two components of the conductive adhesive, the particle size distribution of the AgNPs and WPU_[Cl−]_ were compared (Figure 2b,c) and it was found that AgNP and WPU_[Cl−]_ emulsions are monodisperse; their respective average particle sizes are 30 nm and 120 nm. The WPU_[Cl−]_ molecular chain in the aqueous solution indicates the state of the threads of the entanglement; when WPU_[Cl−]_ dispersion is mixed with alcohol, the viscosity of the WPU_[Cl−]_ dispersion reduces and the molecular chains become loose. Since AgNPs are pre-dispersed in alcohol solvents and the particle size is smaller than that of WPU_[Cl−]_ emulsions, AgNPs can form a homogeneous miscibility with the loose WPU_[Cl−]_molecular chains in alcohol solutions. To further elucidate the interaction between AgNPs and WPU_[Cl−]_in conductive adhesives, the FTIR of WPU_[Cl−]_and conductive adhesives were compared (Figure 2c). The stretching vibration attributed to C=O and C-N on the molecular chain of WPU_[Cl−]_ is red-shifted from the original 1726 cm^−1^ and 1263 cm^−1^ to 1700 cm^−1^ and 1233 cm^−1^, respectively. This indicates that AgNPs can be dispersed more equally in WPU_[Cl−]_with the help of this interaction [40]. In addition, from the XPS spectra of the conductive adhesive (Figure 2e), the 3d5/2 and 3d3/2 electron-orbital binding energy of Ag in the conductive adhesive appeared at 367.5 eV and 373.6 eV, respectively, which are smaller than those of the standard Ag^0^ (368 eV and 374 eV), confirming the interaction between the WPU_[Cl−]_and the AgNPs [41].

Conductive adhesives can be printed on flexible fabric substrates using screen printing. The minimum line width of printing can reach 500 μm, which basically meets the application requirements of printed electrodes (Figure 2f). Further microscopy showed that the boundary of the printed pattern is clear and has no penetration or diffusion (Figure 2g). In addition, the morphology of the PET textile before and after printing was tested and compared by SEM (Figure 2h,i). It was found that the surface of PET fibers before printing is smooth and neat; after printing, the surface of PET fibers is wrapped with a layer of AgNP conductive coating that is homogeneous. The elemental mapping test also confirmed that the coating is AgNP adhesive (see Figure S5); moreover, each PET fiber was completely bonded by conductive adhesive to form an interconnected network.

### 3.3. Mechanism of Chemical Low-Temperature Sintering

**Figure 3:**
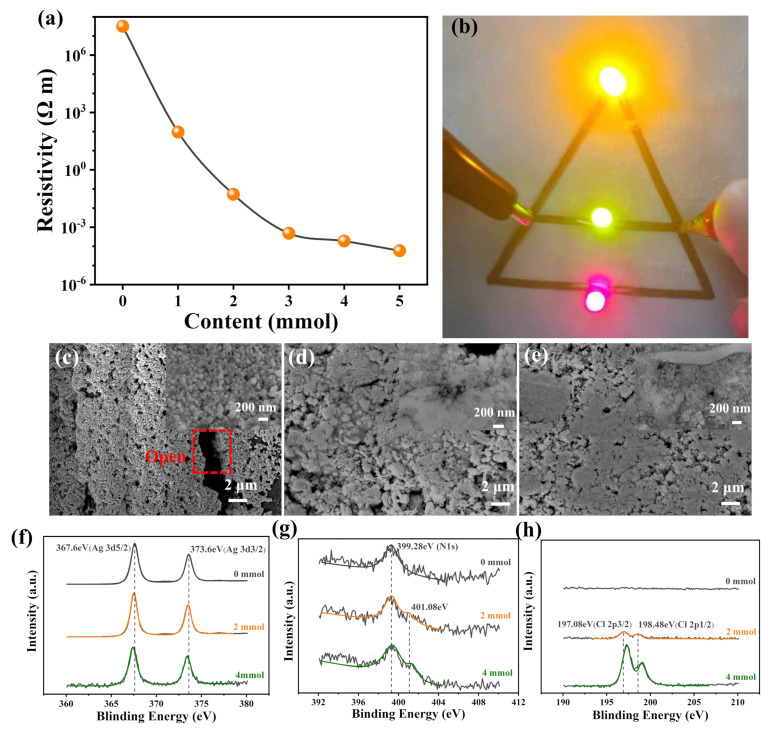
(**a**) Resistivity of as-prepared AgNP-based printed conductive coatings based on different contents of OH-IL_[Cl−]_-OH in WPU_[Cl−]_; (**b**) photo of printed conductive pattern based on 4 mmol of OH-IL_[Cl−]_-OH in a WPU_[Cl−]_application in conductive wire for connecting the LEDs; morphology of printed pattern based on different contents of OH-IL_[Cl−]_-OH in WPU_[Cl−]_: (**c**) 0 mmol, (**d**) 2 mmol, and (**e**) 4 mmol; and XPS spectra of different elements: (**f**) Ag 3d, (**g**) N 1s, and (**h**) Cl 2d.

Conductivity is a core metric for printed circuits [42,43]. It is worth noting that the conductivity of circuits printed from conductive adhesives is related to the amount of OH-IL_[Cl−]_-OH in the WPU_[Cl−]_. When OH-IL_[Cl−]_-OH is not introduced in the WPU_[Cl−]_, the conductivity of the printed circuits is almost close to that of an insulator, giving a 3.1 × 10^7^ Ω m. After the introduction of OH-IL_[Cl−]_-OH, printed patterns can be sintered at low temperatures and are electrically conductive. The conductivity drops dramatically and finally reaches a minimum value of 5.8 × 1 0^−5^ Ω m at an introduction amount of 5 mmol (Figure 3a). The conductivity is also very impressive in comparison with other Ag-based printed circuits (Table S1). In addition, it can be also seen that the conductive Ag-based adhesive made by this method can not only be sintered at lower temperatures to produce low-resistivity printed circuits, but the conductive fillers are also minimized. Based on good conductivity, printed circuits (fabricated from 4 mmol of OH-IL_[Cl]_-OH of AgNP conductive adhesive) at 3 V DC can make the light-emitting diode (LED) operate normally (Figure 3b). Flexible circuits should have high stability and friction resistance. Subsequently, flexible circuits printed on textiles are washed and taped multiple times to evaluate their stability. After 10 washes/tape adhesion, the resistivity of the printed flexible circuits increased from 1.75 10^−4^ Ω m to 3.25 10^−4^ Ω m and from 1.82 10^−4^ Ω m to 2.31 10^−4^ Ω m, respectively (see Figure S6). The high desorption resistance is owing to the ability of the WPU_[Cl−]_ to securely stick the AgNPs to the textile. To confirm, the lap shear test was used to test the adhesion strength of WPU_[Cl−]_to PET-based textile substrates (see Figure S7). Obviously, the adhesion strength of WPU_[Cl−]_to textiles changes with the addition of OH-IL_[Cl−]_-OH, and the adhesion strength (force/width) is as high as 22.5 N cm^−1^ when the OH-IL_[Cl−]_-OH content reaches 2 mmol. Although a decrease in the adhesion strength occurs with the OH-IL_[Cl−]_-OH content exceeding 2 mmol, the adhesion strength decreases at 4 mmol, and thus 17.2 N cm^−1^ is still maintained, which is only a 24% decrease from the highest value. This is mainly due to the fact that the introduction of OH-IL_[Cl−]_-OH increases the polarity of the WPU_[Cl−]_molecular chain favoring the adhesion of the textile substrate; however, the introduction of an excessive amount increases the ionic concentration of the molecular chain, which leads to mutual repulsion weakening intermolecular interactions.

As we all know, electronic conduction is mostly accomplished by contact transmission; therefore, in the composite powder, the conductive components in the powder coupled to one another to form a conductive network are critical [44]. In order to further elucidate the role of OH-IL_[Cl−]_-OH in sintering, their morphology was tested, as displayed in Figure 3c–e. The morphology of AgNPs using WPU_[Cl−]_without OH-IL_[Cl−]_-OH introduction is loose, as can be observed in the high-resolution SEM; these AgNPs are uniformly dispersed in the WPU matrix and have no contact with each other, suggesting that sintering is not occurring (Figure 3c). After the introduction of OH-IL_[Cl-]_-OH into the WPU_[Cl−]_, the AgNPs in the printed circuits begin to undergo the tendency of aggregation, and the presence of a sintered neck can be observed in the high-resolution SEM image (Figure 3d). The AgNPs are divided into solid clumps when the content of OH-IL_[Cl−]_-OH is more than 4 mmol (Figure 3e). Further, elemental mapping of the printed circuits containing 4 mmol of OH-IL_[Cl−]_-OH shows that the elements Ag, C, N, O, and Cl are uniformly dispersed in the printed circuits, and no localized concentration is found (Figure S8). The proportion of Cl in the system is ca. 17.1%, which is in accordance with the optimal concentration reported in the other literature [45]. In order to clarify the mechanism of morphological evolution of the printed circuits under OH-IL_[Cl−]_-OH, XPS was further utilized to resolve the internal chemical structure changes. After the introduction of OH-IL_[Cl−]_-OH into the WPU_[Cl-]_, the binding energies of 196.78 eV and 198.42 eV appeared in the XPS spectra, which are attributed to Cl 2p (see Figure S9). This suggests that Cl^−^ plays an important role in interacting with the AgNPs and stays in the conductive pattern in the printed circuits with the introduction of OH-IL_[Cl−]_-OH. In the Ag 3d orbitals, the binding energies without the introduction of OH-IL_[Cl−]_-OH appear at 367.8 eV and 373.7 eV, respectively, which shift to a lower level after the addition of OH-IL_[Cl−]_-OH, confirming that Cl^−^ interacts with the AgNPs and causes an increase in the negative charge on the surface of the AgNPs (Figure 3f). The same result can also be observed in the Cl 2p orbital, showing that binding energy for Cl shifts to higher energies with increasing OH-IL_[Cl−]_-OH content (Figure 3g). This is mainly due to the strong interaction of Cl^−^ with CA-Na that drives the electron density of Cl. In terms of the N1s orbital, the binding energy appearing only at 399.5 eV in the WPU_[Cl−]_without the introduction of OH-IL_[Cl−]_-OH is attributed to its C-N, the new 401.8 eV after the introduction of OH-IL_[Cl−]_-OH is attributed to the N^+^ on the imidazole ring, and it was found that the characteristic peaks of the C-N binding energy shift to the low binding energy position with the increase in the content, suggesting an increase in the amount of OH-IL_[Cl−]_-OH, and the degree of aggregation between AgNPs is also enhanced (Figure 3h).

### 3.4. Printable AgNP-Based Adhesive for E-Textile Applications

**Figure 4:**
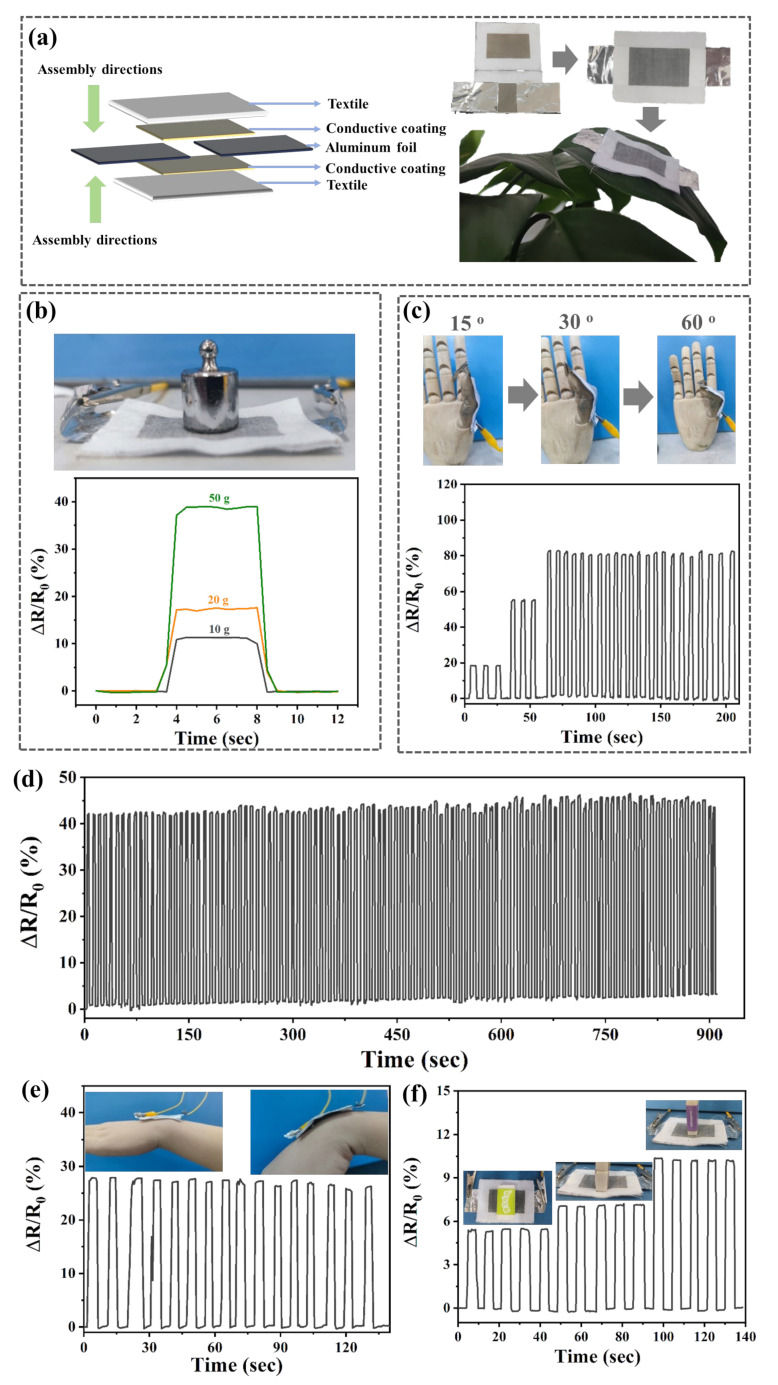
(**a**) Structure and photographs of textile pressure sensors based on AgNP-based conductive adhesive; (**b**) real-time resistance curve of the textile pressure sensor for detection of different weights with 10 g, 20 g, and 50 g; (**c**) real-time resistance curve of the PET-FPS to thumb bending; (**d**) stability of the PET-FPS on weight detection (50 g); (**e**) real-time resistance curve of PET-FPS on monitoring wrist vibration; and (**f**) the PET-FPS on area recognition.

The conductivity of the printed circuit can be adjusted according to the OH-IL_[Cl−]_-OH content, and the printed circuit can be regulated as a semiconductor. Two pieces of PET textile printed with a rectangular conductive pattern (using 2 mmol of OH-IL_[Cl−]_-OH) were assembled in a face-to-face manner to form a PET-FPS (Figure 4a). When an external force is applied to the surface of the PET-FPS, the semiconductor PET textile is forced to squeeze and form contact or separation, causing the contact area of the upper and lower two pieces of the electrode to increase or decrease under the action of the applied pressure, resulting in a significant change in the performance of the pressure response characteristics. Since the PET textile is very lightweight (density of 0.939 g cm^−3^), the PET-FPS can be placed on top of the blades. This also indicates that the assembled PET-FPS has the thinness and lightness of a wearable device. In order to evaluate the sensitivity of the PET-FPS, different masses of weights (10 g, 20 g, and 50 g) were loaded onto the sensors to output corresponding resistance signals; a relationship between the higher mass and the lower resistance was found, and they showed significant resistance variability. Holding the weights on the PET-FPS for a period of time did not reveal excessive drift in its output resistance signal. This indicates that the PET-FPS has a better response to external forces. The stability of the PET-FPS was further measured, and the sensing signals for the PET-FPS were essentially stabilized by repeatedly loading/unloading 100 times with a 50 g weight (Figure 4c). Furthermore, response time is an important parameter to assess the sensitivity of the sensor. A quick thumb-bent test of the PET-FPS showed that the resistance signal is sharp and fluctuates with the bent angles. In addition, from the resistance peak, the width time was deduced in that the frequency of the finger bent is 0.5 Hz (Figure 4d), which indicates that the PET-FPS also has a reliable response performance to the rapid change in pressure. Subsequently, the response time and relaxation time of the PET-FPS were evaluated using the approximate time difference method, and the results indicated times of only 43 ms and 26 ms, respectively (Figure S10). Owing to the excellent responsiveness and reliable signal output of the PET-FPS, it was applied to the monitoring of human joint movement. The sensing signal of the PET-FPS is very stable and reproducible over consecutive flexion–bending cycles of the elbow. Meanwhile, during a bend–hold–recovery process, the PET-FPS fixed to the back of the wrist continuously outputs a step-like sensing signal. Additionally, the PET-FPS is capable of detecting minute pressure changes. As shown in Figure 4f, different bottom areas of a rectangular eraser with the same mass (2.9 g) were loaded onto the PET-FPS (Figure 4f). It was found that their output resistance signals were slightly different, which is primarily due to the erasers having the same mass, but the different PET-FPS bottom areas resulted in the upper and lower surfaces of the PET-FPS contact efficiency being not the same, resulting in a small difference in the resistance signal. These results indicate that the PET-FPS has reliable sensing stability and has potential applications in the development of future smart e-textiles.

**Figure 5:**
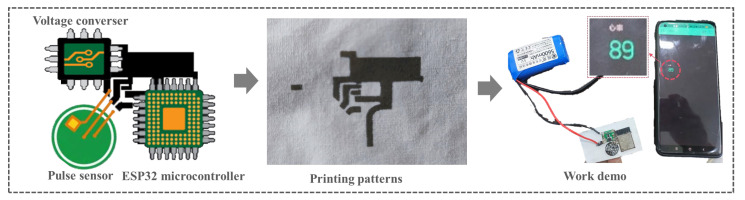
Conductive AgNP-based printed circuits for electrical component integration.

According to the regulation of OH-IL_[Cl−]_-OH on the conductivity of printed circuits, it is known that the minimum resistivity can be as low as 10^−5^ Ω m, which has the potential to be applied as a flexible conductor. We transplanted the components of a commercialized finger pulse device onto a PET textile printed with a designed flexible circuit (using 4 mmol of OH-IL_[Cl−]_-OH, Figure 5). The commercialized finger pulse device consists of an information acquisition module (pulse sensor, 3.3–5 V working voltage), an information processing module (ESP32 microcontroller with Bluetooth, 3.3–3.6 V working voltage), and a voltage conversion module. These components were soldered onto the printed circuit and connected in series. The 5 V power supply is first stabilized by the voltage converter module to output a voltage of 3.7 V to supply the pulse sensor and the microcontroller ESP32 for operation. The pulse sensor is responsible for collecting finger pulse data and outputting it to the microcontroller ESP32, which then collects heart rate data every 10 ms for processing and delivers it to the mobile device via Bluetooth. It was found that the designed printed circuit can successfully connect the components in series and maintain normal operation. Touching the electronic fabric connected to the pulse sensor to the fingertips, the pulse rate recorded on the mobile device was 89 bits, which is comparable to the heart rate of a healthy male of ~25 years of age. This suggests that the printed circuit has great potential for use as a flexible PCB.

## 4. Conclusions

A bis−hydroxy−functionalized imidazolium chloride salt was synthesized using quaternary nitridation reaction and introduced into the molecular chain of WPU by stepwise growth method to form an ionic WPU_[Cl−]_ adhesive. Owing to the strong interaction between Cl^−^ and AgNPs, the stabilizers on the surface can be desorbed to play the role of low−temperature sintering, moreover, the adhesive can firmly adhere the AgNPs to the textile substrate, realizing the low−temperature integrated molding of printed circuits. The Cl^−^ ion concentration can be influenced by the OH−IL_[Cl−]_−OH content thereby modulating the resistivity of the printed circuit. When the resistivity reaches 0.05 Ω m, the printed circuits have the ability of resistive response, which can be assembled into textile pressure sensors that exhibit excellent sensing characteristics, including accurate differentiation of different external pressures (min. 10 g, ~29 Pa), synchronized response to thumb pressure, good stability, and fast response time. In addition, the sensor is capable of detecting joint muscle movements and recognizing the cross−sectional area of light mass objects. When the resistance reaches 5.8 ⅹ 10^−5^ Ω m, the printed circuit shows good conductivity and has the possibility to be used as a flexible PCB. The components of a commercial finger pulsometer can be successfully connected in series and maintained in operation. The versatility of the flexible printed circuits prepared by this ionic WPU_[Cl−]_ has great potential for the development of future e−textiles.

## Data Availability

Data will be made available upon request.

## Supplementary Materials

The following supporting information can be downloaded at: https://www.mdpi.com/article/10.3390/polym16040540/s1, Figure S1: (a) Chemical reaction for synthesis OH-IL_[Cl-]_-OH; (b) ^1^H-NMR spectra of the OH-IL_[Cl-]_-OH; Figure S2: TG curve for AgNPs; Figure S3: XRD spectra of AgNPs; Figure S4: FTIR spectra of WPU_[Cl-]_; Figure S5: Elemental mapping of the printed/unprinted PET: upper is printed circuits; down is unprinted PET textile; Figure S6: Resistivity stability of AgNPs-based printed circuit: (a) cyclic washing treatment; (b) cyclic tape adhesion treatment; Figure S7: Lap-shear measurement for WPU_[Cl-]_ to PET textile with different content of OH-IL_[Cl-]_-OH; Figure S8: Energy spectrum and elemental mapping of the printed circuits based on 4 mmol of OH-IL_[Cl-]_-OH; Figure S9: XPS spectra of AgNPs based on different content of OH-IL_[Cl-]_-OH in WPU_[Cl-]_; Figure S10: Hysteresis curve of PET-FPS; Table S1: Comparison of comprehensive performances of the Ag-based printed circuit.
