# Supplementary material for: Novel Conductive AgNP-Based Adhesive Based on Novel Poly (Ionic Liquid)-Based Waterborne Polyurethane Chloride Salts for E-Textiles
Source: Polymers (Basel). 2024 Feb 17;16(4):540. doi: 10.3390/polym16040540 (PMC10892050; doi:10.3390/polym16040540)
Supplement: Supplementary file 1 [file polymers-16-00540-s001.zip › polymers-2831746-supplementary.pdf]

Novel Conductive AgNP-Based Adhesive Based on Novel Poly (Ionic Liquid)-Based Waterborne Polyurethane Chloride Salts for E-Textiles

Haiyang Liao [1,2], Yeqi Xiao [1], Tiemin Xiao [1], Hongjin Kuang [1], Xiaolong Feng [1], Xiao Sun [1], Guixin Cui [2],
Xiaofei Duan [1] and Pu Shi [1,*]

[1] School of Mechanical Engineering, Hunan University of Technology, Zhuzhou 412007, China; haiyangliao1990@163.com (H.L.); xyqdwyyx@126.com (Y.X.); 18374273723@163.com (T.X.); kuanghongjin999@163.com (H.K.); 15918831619@163.com (X.F.); sxbug@163.com (X.S.); dxf0923@163.com (X.D.)
[2] China Textile Academy (Zhejiang) Technology Research Institute Co., Ltd., Shaoxing 312071, China; cuiguixin3@163.com
* Correspondence: shipu@hut.edu.com

Supporting information:

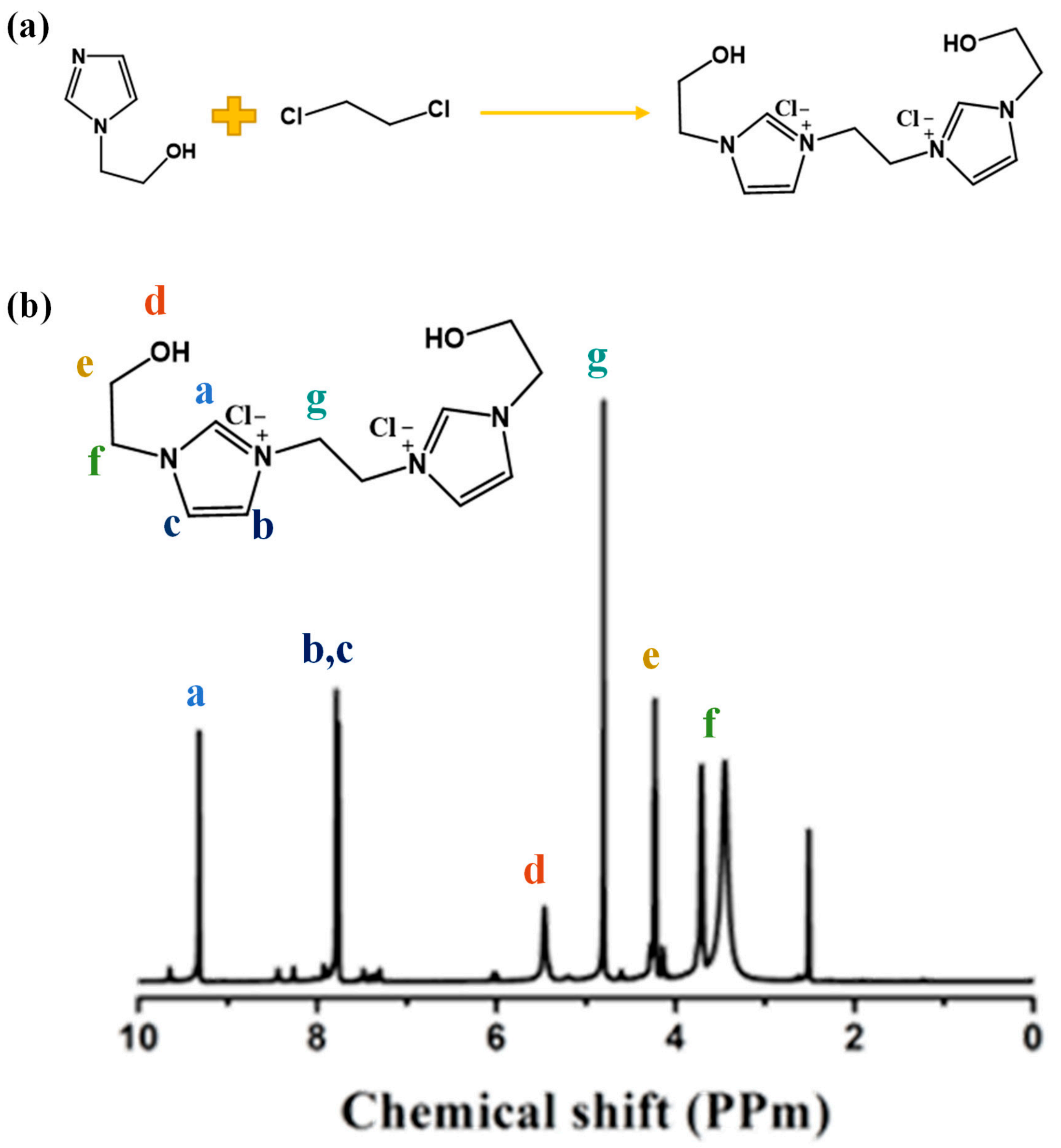

Figure S1. (a) Chemical reaction for synthesis OH-IL$_{[Cl-]}$-OH; (b) ^{1}H-NMR spectra of the OH-IL$_{[Cl-]}$-OH

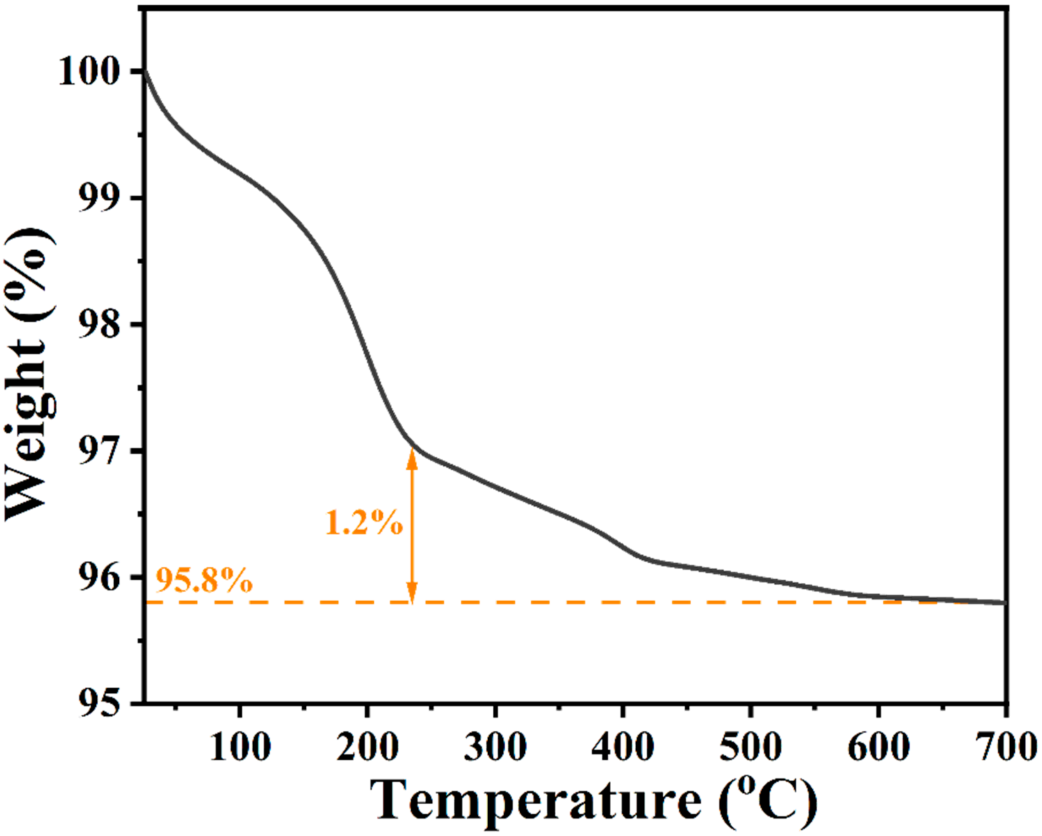

Figure S2. TG curve for AgNPs

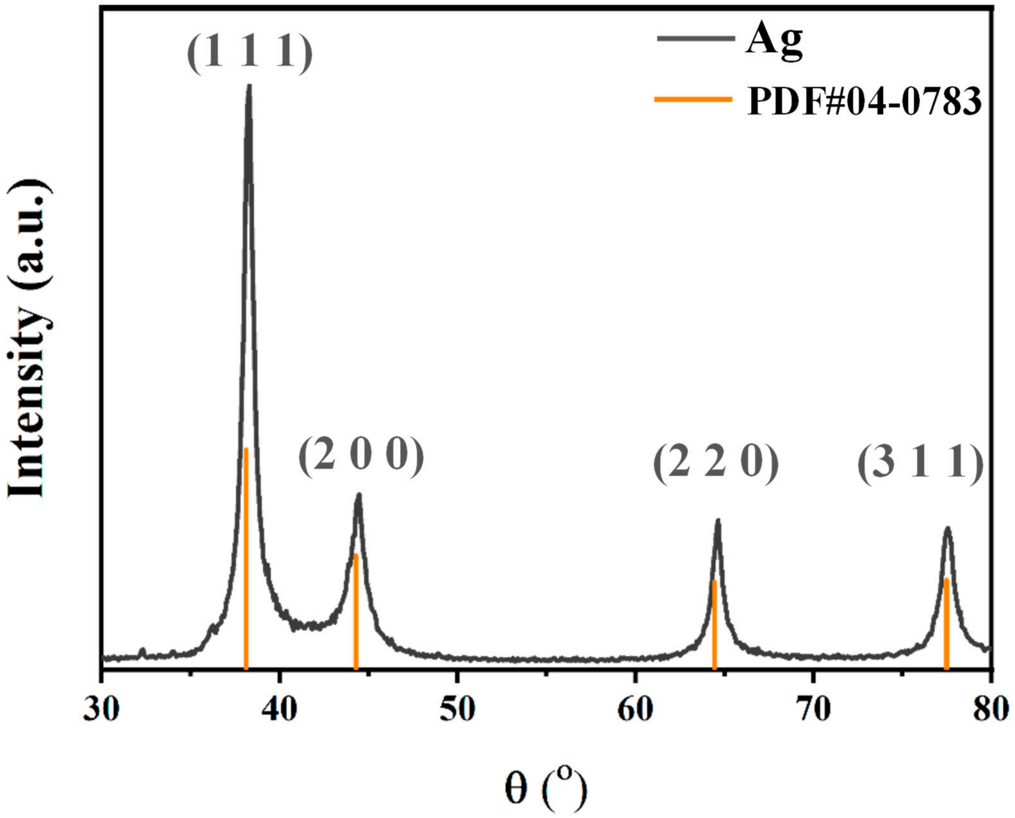

Figure S3. XRD spectra of AgNPs

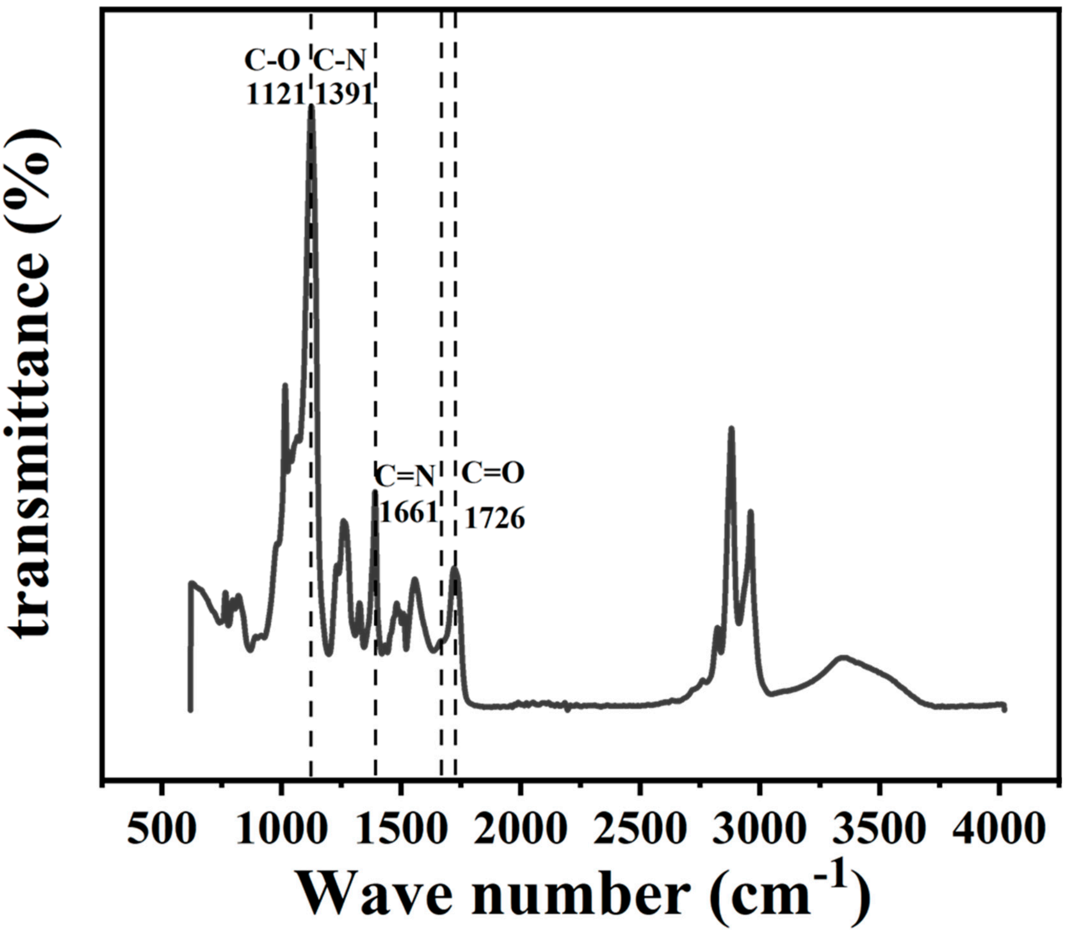

Figure S4. FTIR spectra of $WPU_{[Cl-]}$

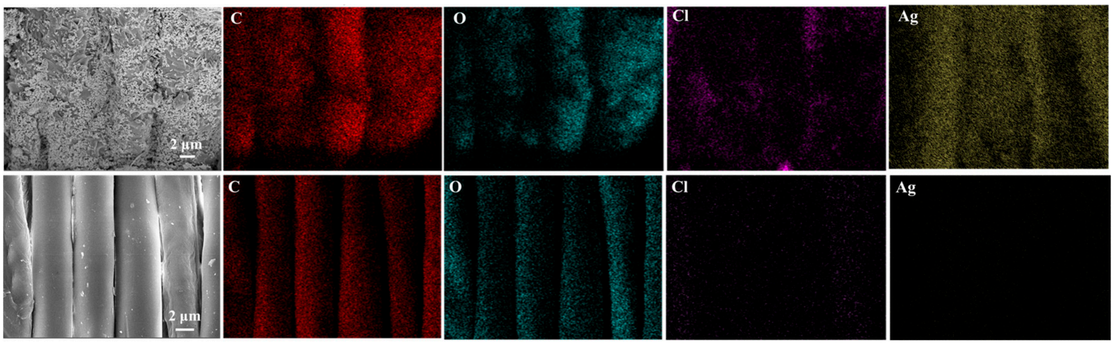

Figure S5. Elemental mapping of the printed/unprinted PET: upper is printed circuits; down is unprinted PET textile

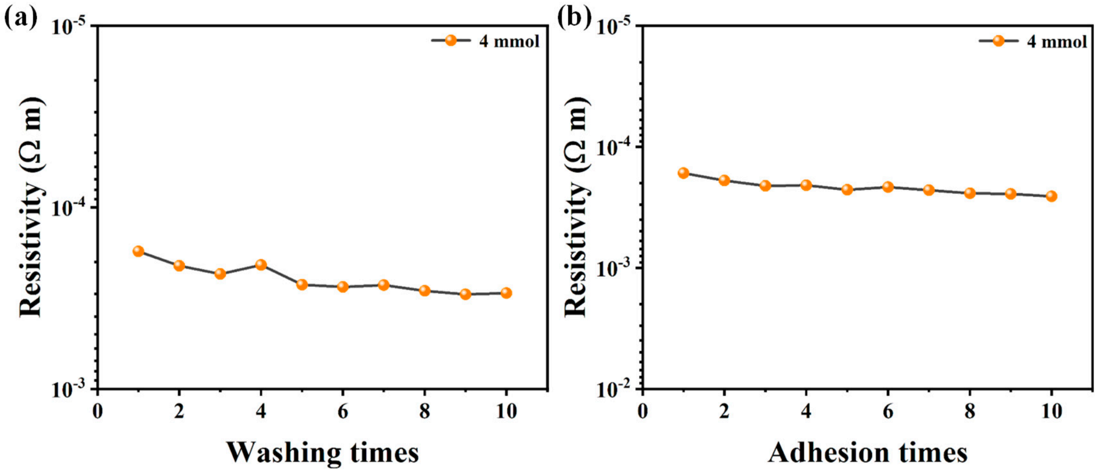

Figure S6. Resistivity stability of AgNPs-based printed circuit: (a) cyclic washing treatment; (b) cyclic tape adhesion treatment

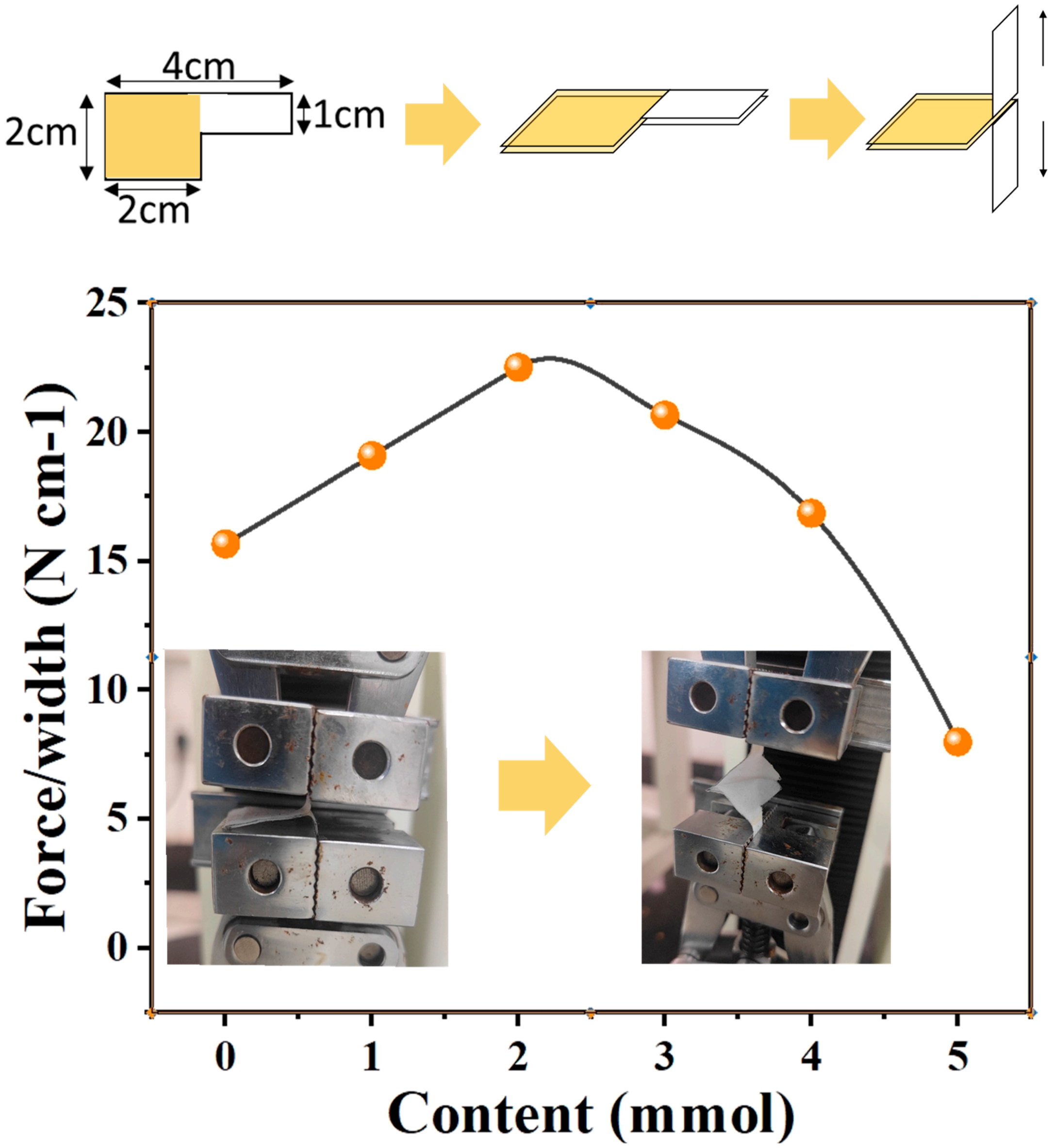

Figure S7. Lap-shear measurement for $WPU_{[Cl-]}$ to PET textile with different content of OH-$IL_{[Cl-]}$-OH

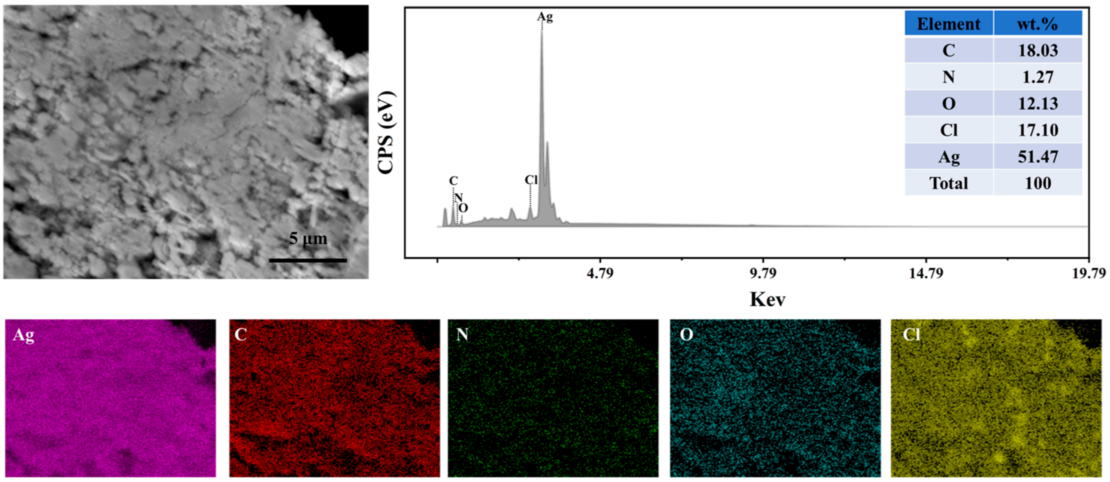

Figure S8. Energy spectrum and elemental mapping of the printed circuits based on 4 mmol of OH-IL$_{[Cl-]}$-OH

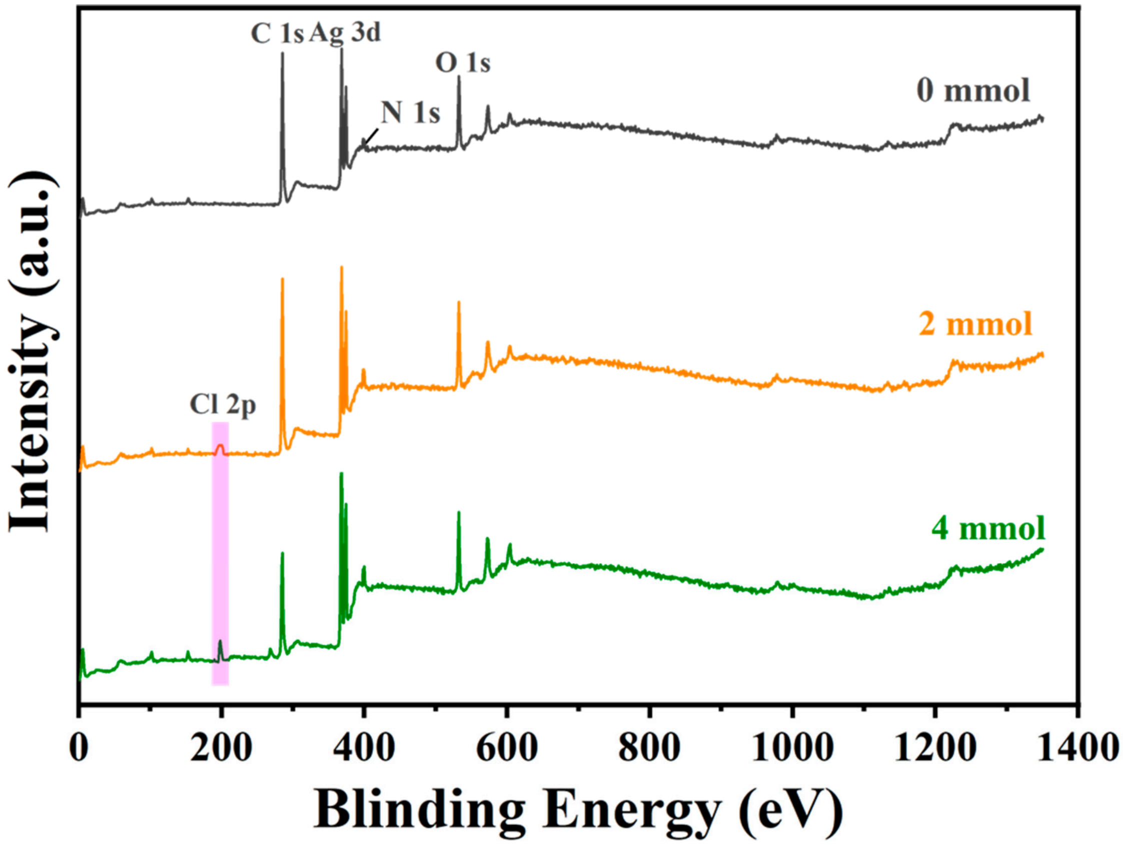

Figure S9. XPS spectra of AgNPs based on different content of OH-IL$_{[Cl-]}$-OH in WPU$_{[Cl-]}$

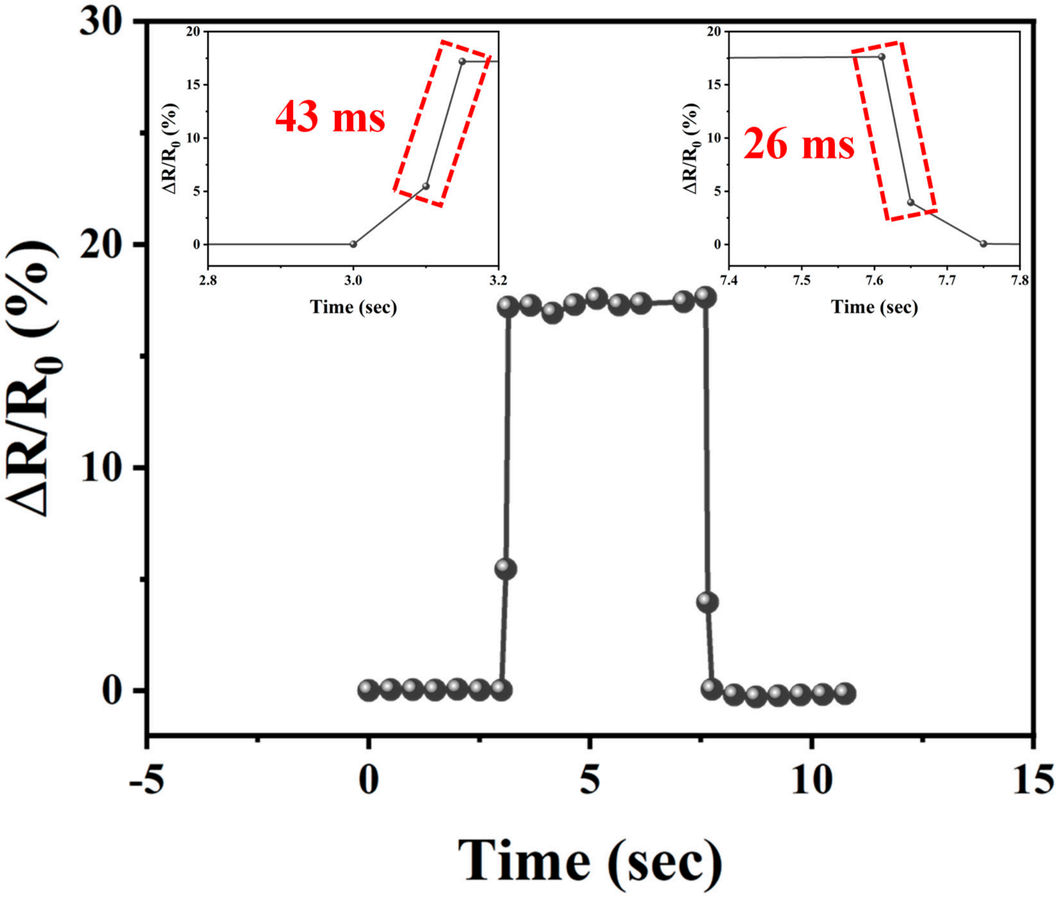

Figure S10. Hysteresis curve of PET-FPS

Table S1. Comparison of comprehensive performances of the Ag-based printed circuit

Sample name	Sintering Temp.	Mass ratio of AgNPs	Resistivit y	Ref.
AgNPs adhsive	60 °C	60%	10^{-5} Ω m	This work
Ag flakes/nAg-MWNTs/NBR	170 °C	80%	10^{-3} Ω m	Ref. S1
Ag flakes/PDMS	160 °C	80%	10^{-4} Ω m	Ref. S2
Ag nanoflowers/PU	155 °C	86%	10^{-4} Ω m	Ref. S3
Ag nanowires inks	150 °C	100%	10^{-5} Ω m	Ref. S4
Ag flakes ink	160 °C	100%	10^{-2} Ω m	Ref. S5
Ag flakes/PI	250 °C	75%	10^{-2} Ω m	Ref. S6
Ag flakes/graphene/epoxy	150 oC	65%	10^{-3} Ω m	Ref. S7

References

S1 S. Kwon, R. Ma, U. Kim, H. R. Choi, S. Baik, Carbon, 2014, 68, 118-124.

S2 Z. Li, T. Le, Z. Wu, Y. Yao, L. Li, M. Tentzeris, K.-S. Moon, C. P. Wong, Adv. Funct. Mater., 2015, 25, 464-470.

S3 R. Ma, B. Kang, S. Cho, M. Choi, S. Baik, ACS Nano, 2015, 9, 10876-10886.

S4 L. Jiajie, T. Kwing, P. Qibing, Adv. Mater., 2016, 28, 5986-5996.

S5 W. Li, X. Xu, W. Li,Y. Zhao, M. Chen, J. Mater. Sci., 2018, 53, 6424-6432.

S6 B. G. Park, K. H. Jung, S. B. Jung, J. Alloys Compd., 2017, 699, 1186-1191.

S7 X. Peng, F. Tan, W. Wang, X. Qiu, F. Sun, X. Qiao, J. Chen, J. Mater. Sci.: Mater. Electron., 2014, 25, 1149-1155.
